# A regional population‐based hereditary breast cancer screening tool in Italy: First 5‐year results

**DOI:** 10.1002/cam4.2824

**Published:** 2020-02-11

**Authors:** Laura Cortesi, Bruna Baldassarri, Stefano Ferretti, Elisabetta Razzaboni, Mariangela Bella, Lauro Bucchi, Debora Canuti, Pierandrea De Iaco, Giorgio De Santis, Fabio Falcini, Vania Galli, Lea Godino, Maurizio Leoni, Anna Myriam Perrone, Marco Pignatti, Gianni Saguatti, Donatella Santini, Priscilla Sassoli de'Bianchi, Federica Sebastiani, Mario Taffurelli, Giovanni Tazzioli, Daniela Turchetti, Claudio Zamagni, Carlo Naldoni

**Affiliations:** ^1^ Department of Oncology and Haematology Azienda Ospedaliero Universitaria di Modena Ospedale Civile di Baggiovara Modena Italy; ^2^ Department of Health and Welfare Emilia‐Romagna Region Bologna Italy; ^3^ Medical Oncology Unit University Hospital of Parma Parma Italy; ^4^ Romagna Cancer Registry Istituto Scientifico Romagnolo per lo Studio e la Cura dei Tumori (IRST) IRCCS Meldola Italy; ^5^ Cancer Screening Unit Local Health Agency of Romagna Rimini Italy; ^6^ Unit of Oncologic Gynecology Department of Obstetrics and Gynecology University of Bologna Hospital of Bologna Sant'Orsola‐Malpighi Polyclinic Bologna Italy; ^7^ Division of Plastic Surgery University of Modena and Reggio Emilia Modena Italy; ^8^ Azienda Usl della Romagna Forlì Italy; ^9^ AUSL Modena Mammography Screening Centre Modena Italy; ^10^ Department of Medical and Surgical Sciences University of Bologna Hospital of Sant'Orsola‐Malpighi Polyclinic Bologna Italy; ^11^ Oncology Unit Ospedale Santa Maria delle Croci Ravenna Italy; ^12^ Senology Unit Bellaria Carlo Alberto Pizzardi Hospital Bologna Italy; ^13^ Sant'Orsola‐Malpighi Polyclinic University of Bologna Hospital of Bologna Bologna Italy; ^14^ Department of the Health of Woman, Child and Urological Diseases University of Bologna Hospital of Bologna Sant'Orsola‐Malpighi Polyclinic Bologna Italy; ^15^ Department of Medical and Surgical Sciences for Children and Adults University of Modena and Reggio Emilia Modena Italy; ^16^ Department of Hematology and Oncology University of Bologna Hospital of Bologna Sant'Orsola‐Malpighi Polyclinic Bologna Italy

**Keywords:** hereditary breast ovarian cancer, population‐based screening, Tyrer‐Cuzick model

## Abstract

**Background:**

Up to 10% of individuals with breast cancer (BC) belong to families with hereditary syndromes. The aim of this study was to develop an instrument to identify individuals/families at high‐hereditary risk for BC and offer dedicated surveillance programs according to different risks.

**Methods:**

The instrument consisted of a primary questionnaire collecting history of BC and ovarian cancer (OC). This questionnaire was applied to women enrolled in the Emilia‐Romagna Breast Cancer Screening Program. General practitioners (GPs) and specialists could propose the same questionnaire too. Women with a score of ≥ 2, were invited to complete an oncogenetic counseling. According to the Tyrer‐Cuzick evaluation, women considered at high risk were invited to involve the most representative alive individual of the family affected with BC/OC for *BRCA1/2* genetic testing.

**Results:**

Since January 2012 and December 2016, 660 040 women were evaluated by the regional screening program, of which 22 289 (3.5%) were invited to the Spoke evaluation, but only 5615 accepted (25.2%). Totally, also considering women sent by GPs and specialists, 11 667 were assessed and 5554 were sent to the Hub evaluation. Finally, 2342 (42.8%) women fulfilled the criteria for genetic testing, and 544 (23.2%) resulted *BRCA1/2* mutation carriers.

**Conclusions:**

To our knowledge, this is the first regional population‐based multistep model that is aimed to identify individuals with *BRCA1/2* mutations and to offer an intensive surveillance program for hereditary‐high risk women. This tool is feasible and effective, even if more efforts must be performed to increase the acceptance of multiple assessments by the study population.

## Graphical abstract

This is, to our knowledge, the first multistep approach, including regional breast cancer screening program, general practitioners and specialists, aimed to identify individuals with BRCA1/2 mutations and to offer an intensive surveillance program for hereditary, high and intermediate risk women. Our results show that a regional population‐based model is feasible, even if more efforts have to be yielded to increase the acceptance of subsequent assessments from women at risk.

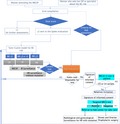

## 1 INTRODUCTION

Hereditary breast ovarian cancer (HBOC) can be defined as a genetic disorder in which breast and ovarian malignant tumors seem to cluster within families.^1^ The main factors that suggest a hereditary cancer predisposition syndrome are young age at cancer diagnosis, multiple tumors, bilateral tumors, presence of rare tumors, several cancer‐affected relatives, autosomal dominant inheritance and, in some cases, ethnicity.^2^, ^3^, ^4^, ^5^, ^6^, ^7^ Families with these characteristics should be referred to specialized hospital/centers that offer cancer risk assessment and genetic counseling^8^, ^9^, ^10^

In Italy, most cancer genetic services are largely distributed in the country but they are not regulated by the national health system rules.^11^

Therefore, there is limited knowledge regarding the prevalence of HBOC predisposition syndromes in the Italian population, which has already reached more than 60 million people.^12^ Moreover, considering the high probability of developing cancer in individuals with hereditary cancer predisposition and the fact that presymptomatic identification of at‐risk individuals offers enormous potential for reducing cancer‐related risk,^13^ a better understanding of the prevalence of hereditary cancer predisposition syndromes in Italy is imperative.

In this context, the goal of this study was to develop a pilot instrument for identifying individuals and families who are at risk for hereditary BC in a regional screening population‐based sample from Emilia‐Romagna, Italy.

## 2 PATIENTS AND METHODS

### 2.1 Ethical aspects

Our program was approved and deliberated by the Emilia‐Romagna region under the protocol number RER 220/2011.^14^ Furthermore, an informed consent form was signed by each woman who participated in the study for the genetic testing analysis and it was approved by the Ethics Committee of each Hub center.

### 2.2 Population of the study

Since January 2012 until December 2016, the regional program for HBOC started in Emilia‐Romagna. Women attending the Regional Breast Cancer Screening Program (RBCSP), which begins at 45 years with an annual mammogram until 50 years and then continues until 74 years with biyearly mammogram, were interviewed by a questionnaire regarding their personal or family history of BC and OC from radiologist technicians. The interview was repeated every screening round to verify changes in the family history. At January 2019, women living in Emilia‐Romagna region, aged between 45 and 74 years, are 960 792. About 69% of them adhere to the RBCSP that is distributed in 8 different units along the region.^15^

Women who asked for their risk at the General Practioner (GP) received the same questionnaire too. Finally, also some specialists, the gynecologists, oncologists, and radiologists, could administer the same questionnaire in the case of positive family history.

### 2.3 Primary screening questionnaire

The primary questionnaire was based on typical criteria for hereditary breast and ovarian cancer, that is, young age at onset, bilateral breast cancer (BC), association with ovarian cancer (OC) and relationship with other affected patients. These criteria were already considered as pathognomonic of hereditary breast cancer syndrome by Lynch.^16^ The questionnaire contained a grid that assigned a score from 0 to 2; women who reached the total score of ≥ 2 were invited to ask for the Spoke evaluation. The grid was adopted since 2000, by the Biosciences Laboratory of Istituto Scientifico Romagnolo per lo Studio e la Cura dei Tumori (IRST), in Meldola, identifying 22% of patients with BRCA1/2 mutations among families with characteristics of hereditary breast and ovarian cancer.^17^ This rate was considered cost‐effective as indicated by the ASCO guidelines.^18^

In addition, a personal or family history of male BC, of BC and OC in the family or in the same patient, of early onset BC (≤35 years), of bilateral BC at ≤50 years, of not mucinous and not borderline sporadic OC, of two first‐degree relatives affected by BC, of which one arisen at ≤40 years or bilateral, and of triple negative BC (≤ 60 years), represented a direct criterion for Hub evaluation. The majority of the primary questionnaires was administered by the radiology technicians at the single unit of the RBCSP, uploading the grid score whenever the patient entered into the mammography room. All questionnaires were collected on a dedicated software program that calculates the score in real time and collects the results on a specific single database for each patient. Also, GPs had the same grid uploaded on their regional electronic health system and autonomously calculated the score when the patient was visited for the first time. The grid was also held by other specialists such as gynecologists, oncologists, and radiologists, but they rarely offered this evaluation to their patients. No informed consent was signed at this time. No central evaluation was performed to calculate the score obtained by different patients.

The questionnaire is shown in Table 1.

**Table 1:** Family risk evaluation

Age at Onset	BC					OC
	<40 y	40‐49 y Bilateral	40–49 y Monolateral	50‐59 y	>60 y	Every
Woman	2	2	1	1	0	2
Mother	2	2	1	1	0	1
Sister	2	2	1	1	0	1
Daughter	2	2	1	1	0	1
Paternal Grandmother	2	2	1	1	0	1
Paternal Aunt	2	2	1	1	0	1
Maternal Grandmother	1	1	1	0	0	1
Maternal Aunt	1	1	1	0	0	1
Relative with MBC	2	2	2	2	2	‐
Cousin (daughter of father's brother)	1	0	0	0	0	1
Nephew	1	1	1	0	0	1

Abbreviations: BC, breast cancer; MBC, male breast cancer; OC, ovarian cancer.

### 2.4 Spoke model

All healthy women with score ≥ 2 or patients affected with BC at the age ranging between 36 and 40 years were invited to refer to their own Spoke center. In case of women attending the RBCSP eligible for further assessment, they received a letter with the mammography result and the phone number for calling the own Spoke center. In case of eligibility defined by GPs or other specialists, the appropriate Spoke center phone number was directly given to the patient. Totally 13 centers in Emilia‐Romagna were accredited as Spoke centers. At the Spoke Center, either geneticists or oncologists trained in oncogenetic counseling collected information on the cancer family history and drawn the pedigree. In both cases a specific informed consent was obtained aimed to receive family history information. The risk of BC was calculated with the Tyrer‐Cuzick program.^19^

### 2.5 Tyrer‐Cuzick risk calculation

The Tyrer‐Cuzick evaluation collects personal information regarding woman's age, menarche's age, height and weight measurements, parity, previous breast lesions, and menopausal status. Further information with regard to family history of BC or OC in the relatives is also collected. The final calculation provides the 10‐year risk and the life‐time risk for BC in individual and general population, respectively.

Women with a life‐time risk of developing BC at most 1 time more than general population were considered at low risk (LR) and were offered RBCSP. Women with a risk ranging between two and three times more than general population, were considered at intermediate profile and followed with an annual mammogram, associated with ultrasound, in the case of dense breasts since 40 until 45 years and then referred to the RBCSP. Women with a risk more than three times (profile 3 or high) were evaluated for referring to the Hub center with the aim to perform gene test analysis.

This model assessment was chosen among different risk calculators, that is, Gail, Claus, Ford, and manual model. It looked to be likely the most sensitive model to select women at high risk, apart from the lack of male breast cancer family history evaluation.^20^ However, women who were sent to the Hub center were further evaluated for BRCA1/2 genetic testing according to the Modena criteria.^21^

### 2.6 Hub model

Four regional Hub centers were accounted in Emilia‐Romagna. The referral to the Hub centers was proposed in the case of profile 3 at the Spoke evaluation or in the case of direct criteria at the primary screening, as previously mentioned.

At the Hub evaluation, a family history of:

1. Early Onset BC
2. BC and OC in the same patient or in other members
3. Male BC
4. Triple Negative BC at ≤60 years
5. OC not mucinous not borderline
6. Two or more BC in the family with a first‐degree relationship each other and young age at onset (≤40 years) or bilateral BC

was considered eligible for genetic testing and the most indicative affected patient in the family was invited to undergo pretest oncogenetic sessions, aimed to perform *BRCA1/2* analysis. In a period of 3‐4 weeks, the *BRCA1/2* results were released along with a posttest genetic session. When a positive test was found, relatives of the index case were invited to undergo specific mutation analysis. All positive women already affected or not, were offered a screening protocol with six‐monthly breast ultrasound since 18 to 69 years, annual breast MRI since 25 until 74 years, annual mammogram since 35 to 69 years and biennial in the 70‐74 years range. In the case of bilateral mastectomy, only breast ultrasound every six months was proposed since the residual cancer risk remains equal to 4%‐5% and no guidelines are provided for breast MRI screening utility in this group of women. Breast ultrasound seemed to have a good ratio between cost and effectiveness rather than a solely clinical examination in order to recognize little foci of cancer upon the protheses. For OC screening, a six‐monthly transvaginal ultrasound plus Ca.125 marker dosage was proposed, when patients refused to consider the prophylactic oophorectomy or when they were too young to offer. The six‐monthly transvaginal ultrasound plus Ca.125 marker dosage was offered on the basis of data obtained by a phase II study in which OC screening performed more frequently than annually with prompt surgical intervention seemed to offer a better chance of early‐stage detection in high‐risk women.^22^ In the case of previous OC, only Ca.125 marker dosage every six months was offered.^14^

**Figure 1:**
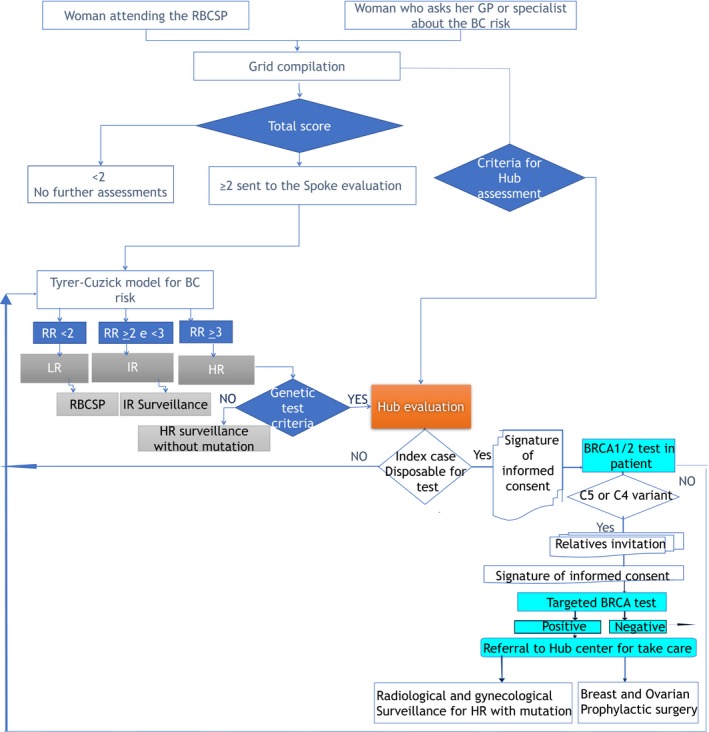
The flowchart of patients through the Spoke and Hub screening procedure. BC, breast cancer; GP, general practitioner; HR, high risk; IR, intermediate risk; LR, low risk; RBCSP, Regional Breast Cancer Screening Program

However, when criteria or individual disposable for gene testing lacked, a dedicated screening protocol was offered to women with different risk profiles; in the case of high risk (profile 3) it starts at 25 years of age with six‐monthly ultrasound until 49, annual mammogram since 35 to 69 years and then biennial until 74 years. For LR and intermediate risk (IR), the screening protocol was described above.^14^ The surveillance protocol is detailed in Table 2. The process mapping of patients through Spoke and Hub screening procedure is shown in Figure 1.

**Table 2:** Surveillance program for different risk profiles

Risk profile	Start	US	MX	MRI
Profile 1	45 y	If suspected mammogram image	45‐50 y A^a^ 51‐74 y B^b^ (Population Screening)	
Profile 2	25 y (if relative with EOBC^c^) 36 y	>41 y if high breast density or suspected mammogram image	40‐50 y A^a^ 51‐74 y B^b^ (Population Screening)	According to EUSOMA guidelines
Profile 3 (without detected mutations)	25 y	25‐60 y S^d^	35‐69 y A^a^ 70‐74 y B^b^	According to EUSOMA guidelines
Profile 3 (with detected mutations)^e^	From the mutation detection	From the mutation detection‐69 y S^d^	35‐69 y A^a^ 70‐74 y B^b^	>25 y A

a Annual

b Biennial

c Early Onset Breast Cancer

d Semestral

e Ca.125 and Transvaginal Ultrasound every 6‐months was also added

### 2.7 Genetic testing

Genetic testing for identifying BRCA1 and BRCA2 mutations included the Next Generation Sequencing (NGS) on the entire codifying sequence and the Multiplex Ligation Polymerase Analysis (MLPA) for rearrangements of BRCA1/2 gene. The variants were divided, according to the ENIGMA classification, in five classes: C5 and C4 were considered as positive results and carriers were followed as previous reported. In the case of C3, C2, and C1 results, the screening was based on the risk profile defined by the Tyrer‐Cuzick risk model. For the C3 classes, a customized pipeline for variant calling was questioned every month in order to find an eventual reclassification.

## 3 RESULTS

### 3.1 Primary screening questionnaire

From January 2012 to December 2016, among 660 040 women participating in the RBCSP, 659 747 women answered the questionnaire, whereas 293 (0.04%) refused to compile the same. Totally, 22 289 women (3.5%) received the letter and were invited to refer to the own Spoke center. By dividing all women in quintiles for age from 45 to 74 years, 8518 of those invited to call on the Spoke evaluation ranged between 45 and 49 years (38.2%). Detailed information on classes of age, number, and percentage of patients referred to the Spoke center by RBCSP can be found in Table 3.

**Table 3:** Women refer to Spoke evaluation by RBCSP according to the age

Total No	Age	%
8518	45‐49	38.2
3836	50‐54	17.2
2856	55‐59	12.8
2511	60‐64	11.3
2354	65‐69	10.6
2214	70‐74	9.9
22 289	All	100

### 3.2 Spoke evaluation

Among all, 22 289 women were referred to the Spoke by the RBCSP, only 5615 (25.2%) phoned for having a Spoke evaluation. All requests were held in about 2 weeks. In addition, 2258 (19.4%) women were referred by GPs and 3794 (32.5%) by specialists to the Spoke centers, with the remaining women (48.1%) coming from RBCSP. Women referred by GPs were, in about one‐quarter of cases, younger than 35 years of age (560/2258, 24.8%), whereas specialists identified more patients in the age 40‐44 years (887/3794, 23.4%). Totally, 11 667 women arrived at the Spoke centers. Among those, 330 refused to complete the assessment, and the remaining 11 337 women were evaluated. Totally, 4627 women evaluated were considered eligible for the Hub referral (40.8%), whereas 6710 (59.2%) had an IR or LR profile. Data on source, age and risk profile are reported in Table 4.

**Table 4:** Characteristics of women arrived at the Spoke centers

Age	GPs	Specialists	RBCSP	Total	%	Refuse	LR	IR	HR
<35	560	840	0	1400	12	32	237	424	707
35‐39	403	722	0	1125	9.6	26	191	365	543
40‐44	509	887	49	1445	12.4	35	309	453	648
45‐49	246	432	1832	2510	21.5	96	835	663	916
50‐54	214	369	1153	1736	14.9	48	612	423	653
55‐59	140	234	861	1235	10.6	28	458	260	489
60‐64	100	146	743	989	8.5	26	409	234	320
65‐69	65	102	599	766	6.6	30	339	180	217
70‐74	21	62	378	461	4	9	210	108	134
Total	2258	3794	5615	11 667	100	330	3600	3110	4627

Abbreviations: HR, High Risk; IR, Intermediate Risk; LR, Low Risk.

Among all 11 667 women who made an appointment at the Spoke center, 1400 (12%) were very young (<35 years), 2570 (22%) ranged between 35 and 44 years of age, whereas 7697 (66%) were aged from 45 to 74 years. Out of 3970 women aged less than 45 years, 2449 (61.7%) were sent at the Spoke Center by specialists, 1472 by GPs (37.1%) and 49 by RBCSP (1.2%).

### 3.3 Hub evaluation

Out of 4627 women assessed as HR (profile 3), only 2815 (60.8%) accepted the Hub evaluation. The most proportion of women eligible for the Hub evaluation was identified by GPs (1205/2241, 53.8%), followed by specialists (1768/3684, 48.0%) and finally by RBCSP (1654/5412, 30.6%) as shown in Table 5.

**Table 5:** Characteristics of women assessed at the Hub centers

	<35	Hub	%	35‐44	Hub	%	45‐74	Hub	%	Total	Hub referral	%
GPs	556	289	52.0	909	473	52.0	776	443	57.1	2241	1205	53.8
Specialists	812	418	51.5	1552	699	45.0	1320	651	49.3	3684	1768	48.0
RBCSP	0	0	0	48	19	39.6	5364	1635	30.5	5412	1654	30.6
Total	1368	717	52.4	2509	1191	47.5	7460	2729	36.6	11 337	4627	40.8

Additionally, following the seven criteria considered as enough for to the Hub referral at the primary questionnaire, 2739 women were sent directly, without the Spoke assessment. Totally, 5554 women received a Hub evaluation.

**Figure 2:**
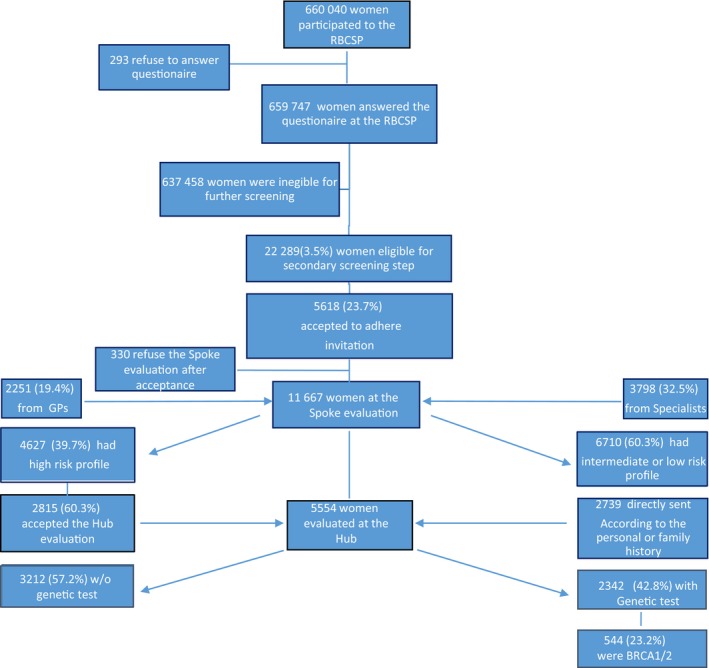
Number of patients evaluated along the process mapping

In 2342 cases (42.2%), a genetic test was performed, whereas 3212 (57.8%) women did not meet criteria for *BRCA1/2* analysis. Five hundred and forty‐four women (23.2%), resulted *BRCA1/2* mutation carriers. As expected, among *BRCA1/2* mutated women, the highest rate of mutations was found in very young women, aged less than 30 years, (35%); the positive rate decreased until 18% in women ranging between 55 and 59 years. Women without mutation ascertained were classified as having profiles 1, 2, or 3 according to the previous Tyrer‐Cuzick risk calculation. Table 6 reports data on women who underwent a genetic test. The number of patients along the process mapping is depicted in Figure 2.

**Table 6:** Characteristics of women underwent *BRCA1/2* gene analysis

Age	N° LR	% LR	N° IR	% IR	N° HR	% HR	N° BRCA1/2	% BRCA1/2	Total
<25	44	40	23	21	8	7	36	32	111
25‐29	23	23	19	19	24	24	35	35	101
30‐34	44	25	24	14	58	33	49	28	175
35‐39	68	24	38	13	115	41	62	22	283
40‐44	64	21	44	15	115	38	80	26	303
45‐49	83	26	45	14	123	39	66	21	317
50‐54	74	26	24	8	132	46	58	20	288
55‐59	57	26	15	7	107	49	38	18	217
60‐64	41	22	14	8	92	50	37	20	184
65‐69	40	25	3	2	82	51	35	22	160
70‐74	51	25	3	1	101	50	48	24	203
**Total**	**589**	**25.1**	**252**	**10.8**	**957**	**40.9**	**544**	**23.2**	**2342**

## 4 DISCUSSION

Our model is an effective tool to identify individuals/families at risk for BC, in a population‐based sample. In fact, nearly all women attending the RBCSP, with the exception of 293 cases (0.04%), compiled the primary questionnaire, also taking into account the change of family history along time. This multistep tool, proposed by a healthcare staff, provides more awareness about hereditary BC than a recently developed nurse navigator approach, by which a low participation of screening patients requesting HBOC education and evaluation occurred (9%).^23^

Hoskins et al,^24^ previously validated a tool for each case of BC or OC arbitrarily selected, weighting more points for patients with features associated with a higher probability that a *BRCA* mutation is present: early age of BC diagnosis, OC diagnosis, male BC in the family.^25^, ^26^, ^27^, ^28^, ^29^ The authors analyzed a total of 3906 women without a personal history of BC presenting for a screening mammogram at a community hospital, identifying 86 (2.2%) women with a family history indicative of a high probability (>10%) that a BRCA mutation was present. The percentage of at‐risk families was superimposable to our selection model in which 22 289 (3.5%) women were eligible for a secondary screening step.

An added value of this multistep process has been provided by GPs and specialists involvement. A recent systematic review on 40 studies published between 1996 and 2017, has evaluated the effects on patients of genetic cancer risk assessment in general practice.^30^ A variety of testing and screening tools were available for genetic cancer risk assessment in general practice, principally for breast‐ovarian, but GPs often reported low knowledge about hereditary cancers even if, time along, they were increasingly interested. In our experience too, the lowest percentage of women sent to the Spoke evaluation, came from GPs (19.4%) with respect to specialists (32.5%) and RBCSP (48.1%). However, GPs and specialists were needed to identify young high‐risk individuals, since the RBCSP begins at 45 years. In fact, 24.8% of women referred to the Spoke centers by GPs were younger than 35 years, such as women sent by specialists were mostly inclusive between 40 and 44 years (23.4%). As already seen in a recent paper that investigated the young women's perceptions regarding communication with healthcare providers about BC risk, people aged 18‐29 years asked more than doctors about their risk, whereas women in the age group between 30 and 44 years were likely queried by GPs about their family history.^31^ Totally, only 34% of all women evaluated at the Spoke centers aged less than 45 years and this could represent a critical issue, in order to offer a very preventive strategy in young individuals at hereditary‐HR for BC.

Another weakness was shown by the low rate of adhesion to the Spoke evaluation among women eligible by the RBCSP (25.2%). The low rate of adherence to the second screening step could depend by the fact that the invitation was provided by the same letter in which the mammogram result was delivered. Probably by this way, women focused their attention on negative mammogram results rather than on the deepening of their BC risk. An attempt to recover women eligible for the second screening step has been performed at the subsequent round, where the grid was repeated with a little increased rate of acceptance (0.3%). In a Brazilian study performed on 20 000 women attending the mammogram screening program, a questionnaire regarding BC family history was proposed and 3121 (15.6%) were invited to the second phase of the study by a direct approach or by letter or by phone. The first two modalities of invitation provided a very low rate of adhesion (11.4% and 16%, respectively), lower than our rate of acceptance. The highest rate of adherence was reached by the phone call (72.6%), demonstrating the validity for this approach for identifying families at risk for BC.^32^ However, the telephone approach needs dedicated personnel, and expensive costs for a large regional screening program. Also, considering the recall carried out at the subsequent round, we concluded that women who did not attend the Spoke assessment did not want to know their BC risk, with respect to the nondirectiveness defined as procedures that promote and enhance the autonomy and self‐control of patients.^33^

Nevertheless, among 11 337 women who received the Spoke evaluation, about 40% were HR, whereas 60% were considered as IR or LR. All LR (3600, 31.8%) and young IR women (aged 45 years or more, that is, 1868 equal to 16.6%), did not receive any personal prevention screening program but were invited to attend the RBCSP starting at 45 years. Only IR women aged less than 45 years (1294, 11.4%) and all HR individuals (4627, 40.8%) were offered a dedicated screening program for no hereditary risk of BC. Totally, the regional Spoke assessment identified about half of the women (5921, 52.2%) who needed an intensive screening program. A recent analysis performed on 2177 women at HR and IR followed at the Modena spoke center showed a BC detection rate of 8.5 and 16.1 × 1000 persons‐years, respectively, which clearly increases in comparison with the BC detection rate provided by the RBCSP.^34^ This data confirms the usefulness of an intensive screening program for at‐risk women and justifies a massive effort to identify the various parameters.

Once again, only 60% of women sent to the Hub centers accepted to be evaluated, but the rate of adherence was increased with respect to the previous step, underlying more interest toward the knowledge of hereditary conditions. Of notice, 49% of women evaluated at the Hub centers skipped the Spoke assessment according to specific criteria at the primary questionnaire. Among 5554 women evaluated at the Hub centers, 42.8% were eligible for gene testing and 23.2% resulted positive. This multistep approach provides a long patient journey, deriving a very high benefit in terms of BRCA mutation carrier identification. In fact, the step‐by‐step process is able to select a shrinking number of women to be investigated by gene test analysis: this approach looks to be really cost‐effective, although a recent study comparing BRCA1, BRCA2, PALB2, RAD51C, RAD51D, and BRIP1 analysis performed in selected and unselected women, seems to prevent more BC and OC in the general population, without previous selection.^35^ However, our mutation rate is in line with other series based on different eligibility criteria for HBOC, as referred in a recent review where the *BRCA1/2* mutation rate in early onset BC, triple negative BC, bilateral BC, and family history of BC is about 30%.^36^ It also means that direct criteria for the Hub evaluation represent a significant way to collect women who are potentially at risk for HBOC syndrome, avoiding a multistep process and saving time and important costs.

## CONFLICT OF INTEREST

The authors declare that they have no financial disclosure and conflict of interests.

## AUTHOR'S CONTRIBUTION

Laura Cortesi: conceptualization, data curation, formal analysis, writing—original draft, and writing—review and editing. Bruna Baldassarri: conceptualization, funding acquisition, project administration, resources. Stefano Ferretti: funding acquisition, project administration, resources, supervision and writing—review and editing. Elisabetta Razzaboni: conceptualization, data curation, writing—review and editing. Mariangela Bella: data curation, writing—review and editing. Lauro Bucchi: data curation, formal analysis, methodology, and writing—review and editing. Debora Canuti: data curation, formal analysis, and writing—review and editing. Pierandrea De Iaco: conceptualization, data curation, supervision, and writing—review and editing. Giorgio De Santis: investigation, supervision writing—review and editing. Fabio Falcini: conceptualization, data curation, investigation, and writing—review and editing. Vania Galli: data curation, methodology, and writing—review and editing. Lea Godino: data curation, formal analysis, and writing—review and editing. Maurizio Leoni: conceptualization, data curation, formal analysis, supervision, validation, and writing—review and editing. Anna Myriam Perrone: investigation, and writing—review and editing. Marco Pignatti: investigation, and writing—review and editing. Gianni Saguatti: conceptualization, data curation, formal analysis, supervision, and writing—review and editing. Donatella Santini: investigation, and writing—review and editing. Priscilla Sassoli dè Bianchi: data curation, formal analysis, methodology, project administration, software, supervision, validation, and writing—review and editing. Federica Sebastiani: data curation, investigation, and writing—review and editing. Mario Taffurelli: investigation, methodology, and writing—review and editing. Giovanni Tazzioli: investigation, and writing—review and editing. Daniela Turchetti: conceptualization, data curation, formal analysis, investigation, methodology, and writing—review and editing. Claudio Zamagni: investigation, and writing—review and editing. Carlo Naldoni: conceptualization, data curation, formal analysis, funding acquisition, investigation, methodology, project administration, resources, software, supervision, validation, writing—original draft, and writing—review and editing.

## Data Availability

Data available on request due to privacy/ethical restrictions.
